# Determinants of female sterilization method uptake among women of reproductive age group in Uganda

**DOI:** 10.1186/s40834-020-00131-8

**Published:** 2020-10-08

**Authors:** Paula Anita, Abel Nzabona, Richard Tuyiragize

**Affiliations:** 1 School of Statistics and Planning, Makerere University, Kampala, Uganda; 2 Marie Stopes Uganda, Kampala, Uganda

**Keywords:** Female sterilization, Tubal ligation prevalence, Contraceptive decision-making, Uganda

## Abstract

**Background:**

Despite its great effectiveness, safety and convenience for women who do not wish to have more children; female sterilization method uptake in Uganda is very low. This study aimed at establishing factors associated with female sterilization uptake in Uganda.

**Methods:**

Secondary data were sourced from the 2016 Uganda Demographic and Health Survey (UDHS). We analyzed all (18,506) women aged 15–49 years who were interviewed in the 2016 UDHS. This sample was categorized into women who were sterilized and those using other modern methods. We used a Chi-square test to measure the association between the current uptake of female sterilization by the women and selected independent variables. Multivariate analysis applied the complimentary log-log model to determine the net effect of selected characteristics on female sterilization uptake in Uganda.

**Results:**

The overall prevalence of female sterilization among modern contraceptive users was 2%. Female sterilization uptake was highly associated with age of 30 years and older (OR = 34.49;, 95%CI:13.33–99.88), middle wealth status (OR = 0.65, 95% CI:0.47–0.92), women who had ever given birth to at least four children (OR = 3.19, 95% CI:1.63–6.22) and decision making by either the husband/partner (OR = 2.42, 95% CI:1.55–3.78) or jointly between a woman and her husband/partner (OR = 1.38, 95% CI:1.02–1.86).

**Conclusions:**

The uptake of female sterilization was very low, and this was associated with; age, household wealth, parity and contraceptive decision-maker. The uptake of Family planning programs needs to focus on male engagement to increase joint decision making on family planning issues especially those relating to fertility limitation. Government and its other implementing partners need to scale-up efforts that increase accessibility to information on female sterilization services for women who have completed their fertility.

## Introduction

Female sterilization is a permanent contraceptive method used by women who do not wish to have more children. Together with, long-acting methods such as Intra-Uterine Devices (IUDs) and implants, female sterilization (tubal ligation) and vasectomy are the most effective methods of contraception and would thus provide a very safe and convenient alternative [1]. Globally, female sterilization is used by 19% of married women but there are marked regional differences in its uptake among women of childbearing age. Studies show that female sterilization uptake is incomparably high in developed countries.

The prevalence of permanent contraception such as female sterilization in developing countries is only 20.6% [2]. Female sterilization is more common in Asia (23%) and Northern America Oceania and some parts of Asia, but less common in Africa (1.7%) as well as in Central Asia, South-Eastern Asia and Western Asia [3]. In developing countries, about 20 to 30% of women who use oral contraceptives or injectable stop within 2 years of starting due to side effects or other health concerns [4]. The low uptake of female sterilization in sub-Saharan Africa (SSA) is linked to poverty, limited awareness, lack of skilled personnel, limited resources to purchase and maintain sophisticated laparoscopic equipment [5]. In many countries of SSA, fertility rates and unmet need for family planning remain high [6]. Contraceptive prevalence in countries such as Uganda, Ghana, Nigeria and Rwanda among others has been reported to be influenced by partner support, approval or opposition [4].

Despite many women in SSA wanting to stop having children, the proportion using long acting and permanent methods such as female sterilization which is the most effective birth control method is very low [7]. Women and couples could achieve their desired fertility goals by using more effective, reliable and safe methods of family planning. Female sterilization is safe and effectively provides longer protection against pregnancy for women who do not wish to have more children. The 2016 Uganda Demographic and Health Survey (UDHS) report indicated that the proportion of currently married women in Uganda who have undergone sterilization increased from 2% in 2000/01 to only 3% in 2016 [8]. Due to this, women continued to have unwanted fertility as seen in the discrepancy between women’s actual fertility and wanted fertility rate. The 2016 UDHS reports that whereas, total wanted fertility rate for the women in Uganda in the year 2016 was 4.3 children per woman, the actual fertility rate was 5.4 [8]. This implies that women in Uganda have about 1.1 more children than they want. This unwanted fertility can be reduced by using safe, effective methods that have higher continuation rates. Results of the 2016 UDHS indicated that use of modern contraception among currently married women increased from 14% in 2000–01 to 35% in 2016 and injectables remained the most used method [8]. The results also revealed that in the 5 years preceding the survey, 45% of episodes of contraceptive use were discontinued within 12 months and the main reason for discontinuation was method-related health concerns or side effects (35%) [8]. According to the 2016 UDHS report, 28% of currently married women and 32% of sexually active unmarried women have an unmet need for family planning. Due to their effectiveness, reliability, safety and lower (if any) side effects, permanent methods such as female sterilization can improve continuation rates and also reduce unmet need for family planning that may be associated with fears of side effects and method failures.

Most studies in Uganda examined factors that influence uptake of modern contraception methods [6, 9–17]. Some studies also explored the utilization of long acting reversible methods such as implants and intra-uterine device (IUD) [7]. However, there is limited documentation on the non-reversible methods of family planning such as female and male sterilization. Female sterilization contraception method is highly effective (more than 99%), has very low failure rates and minimal serious complications [18] and due to its limited side effects may attract partner approval. Moreover, high female sterilization uptake reduces population growth rate by limiting unplanned births and like other contraceptive methods improves maternal and child health by preventing unwanted, high-risk pregnancies and reducing the need for unsafe abortions [19, 20]. This study explored the factors influencing uptake of female sterilization among women in Uganda.

## Data and methods

Secondary data from the 2016 Uganda Demographic and Health Survey (UDHS) were used. The UDHS is a nationally representative cross-sectional survey on household, socio-demographic, health status and health care data. The survey was conducted by the Uganda Bureau of Statistics (UBOS) with technical support from ICF (originally, Inner City Fund). The UDHS collected data from women, children, men and couples. This study used the women’s dataset which has data on marriage, sexual activity, and the contraceptive behavior of women aged 15–49 years. A total of 18,506 women aged 15–49 years were interviewed during the 2016 UDHS. The samples were obtained using a two-stage cluster sampling process beginning with the selection of clusters followed by the selection of households from each cluster and the selection of respondents from households.

During the survey, the women were asked to report about their current use of any method of contraception to avoid or delay pregnancy. The women were specifically asked; *“Are you or your partner currently doing something or using any method to delay or avoid getting pregnant?* And this led to a follow-up question *“Which method are you using?”* These questions provided responses on various methods used by women to prevent pregnancy which helped to categorize the dependent variable for this study. This study was conducted on all the 18,506 women of reproductive age who were interviewed in the UDHS. Data were weighted to ensure representativeness and control for non-responsiveness across regions. The sample was weighed using the sampling variable and the svy command as recommended by DHS. The weighted sample included 18,165 women who were not sterilized and 341 who were sterilized.

The dependent variable (sterilization status) was generated from the question on current use of family planning methods. We categorized the women into two categories depending on whether they were sterilized or not. The women who were using methods other than female sterilization as well as those who were not using any method of family planning were categorized as “not sterilized”. The independent variables included age, education (highest education level attained by the woman), place of residence, wealth status, marital status, number of children ever born, ideal number of children, ideal number of boys and ideal number of girls, women’s paid employment status, whether woman earns more than partner (based on the question; would you say that the money that you earn is more than what your (husband/partner) earns, less than what he earns, or about the same?), decision maker on respondent’s earnings (who makes the decision on how to spend the woman’s earnings), and contraceptive decision maker (who makes the decision to use contraceptives). Selection of the independent variables was based on previous studies and the hypothetical relationship they have with sterilization uptake. Wealth status was a variable that we generated from the DHS wealth quintiles. In the DHS, wealth quintiles were obtained by using an asset index which grouped households into five categories (poorest, poorer, middle, richer and richest). We combined poorest and poorer to generate the category “poor” and also merged richer and richest categories to generate the “rich” category.

Data were analyzed at three levels using STATA software version 13. We first generated frequency distributions to describe the characteristics of the study sample. Using cross-tabulations and a Pearson’s Chi-square test, we measured the association between female sterilization use and each of the explanatory variables. The complementary log-log model (Clog log) for multivariate analysis. Complementary log-log model is recommended for use in analysis of rare phenomena such as female sterilization use whose prevalence is very low in Uganda. Model adequacy was checked using the link test. All statistical tests were interpreted at a 5% level of significance.

## Results

The analysis was undertaken on a weighted sample of 18,506 women aged 15–49 years. The findings in Table 1 indicate that 60% of the women were younger than 30 years, 61% were currently married, 57% had attained primary level of education and 45% were from rich households. Furthermore, majority of the women (73%) resided in rural areas, 73% were employed and 62% had ever given birth to 0–3 children. Also, majority (81%) of the women preferred at least 4 children, 76% preferred 1–3 boys, and 74% preferred 1–3 girls. It is important to note here that the non-response categories on ideal number of children, ideal number of boys and ideal number of girls refer to women who d made statements such as; “It depends on God”, “As many as I can support”, “I don’t know” rather than specifying numbers when asked about their fertility preferences. Regarding the uptake of female sterilization, Table 1 indicates that only 2% of the women had been sterilized.

**Table 1:** Percentage distribution of women by socio-demographic characteristics (*n* = 18,506)

Characteristic	Frequency (***n***)	Percent (%)
**Age**		
Younger than 30 years	11,137	60.2
30 years and older	7369	39.8
**Marital status**		
Never in Union	4783	25.8
Currently married	11,223	60.7
Formerly married	2500	13.5
**Education level**		
No education	1781	9.6
Primary	10,630	57.4
Secondary+	6095	32.9
**Household wealth status**		
Poor	6643	35.9
Middle	3460	18.7
Rich	8403	45.4
**Place of residence**		
Urban	4943	26.7
Rural	13,563	73.3
**Employment status**		
Not employed	4986	26.9
Employed	13,520	73.1
**Total number of children ever born**		
0–3 children	11,511	62.2
4+ children	6995	37.8
**Ideal number of children**		
None	148	0.8
1–3 children	2937	15.9
4+ children	14,985	81.0
Non-numeric	437	2.4
**Ideal number of boys**		
None	2278	12.3
1–3 boys	14,040	75.9
4+ boys	1658	9.0
Non-numeric	529	2.9
**Ideal number of girls**		
None	2250	12.2
1–3 girls	13,714	74.1
4+ girls	2012	10.9
Non-numeric	529	2.9
**Sterilization status**		
Not sterilized	18,165	98.2
Sterilized	341	1.8
**Total**	**18,506**	**100**

Table 2 indicates that although slightly less than three quarters (74%) of the women earned less than their husband/partner, 53% decided on how to spend their own earnings. The results also show majority of the women (62%) reported that contraceptive decision making was jointly made with their partners. The results also show that 99% had knowledge of any family planning method, 73% did not know the source of any family planning method while 59% obtained family planning methods from a public health facility.

**Table 2:** Distribution of respondents by enabling factors

Factor	Frequency (***n***)	Percent (%)
**Whether respondent earns more than husband/partner (*n*** **= 7096)**		
Earn more than him	671	9.5
Earn less than him	5244	73.9
Earn about the same	925	13.0
Don’t know	256	3.6
**Decision maker on respondent’s earnings (*n*** **= 7096)**		
Respondent	3733	52.6
Husband and wife	2719	38.3
Husband/Partner	644	9.1
**Decision-maker on contraception (*n*** **= 4373)**		
Respondent	1340	30.6
Husband/Partner	312	7.1
Joint decision	2721	62.2
**Knowledge of any family planning method (*n*** **= 18,506)**		
No knowledge	187	1.0
Has knowledge	18,319	99.0
**Knowledge of source of any family planning method (*n*** **= 18,391)**		
Don’t know	13,456	73.2
Public source	2888	15.7
Private source	1914	10.4
Other	133	0.7
**Source of family planning method (*n*** **= 4935)**		
Public facility	2888	58.5
Private facility	1914	38.8
Other	133	2.7

Table 3 presents the distribution of female sterilization by selected characteristics. The results in the table indicate that age, marital status, education level attained, employment status, total number of children ever born, ideal number of children, ideal number of boys, ideal number of girls and whether the husband earned more than the partner/husband were significantly associated with female sterilization uptake (*p* < 0.05). On the other hand, wealth status, place of residence, decision maker on respondent’s earnings, decision maker on contraception and preexisting knowledge of family planning were not significant. The non-numeric responses as presented in the Table on fertility preferences whether it was with ideal number of children, ideal number of sons and daughters are for the proportion of women who did not specify the ideal numbers of children they preferred but rather made statements such as; “It depends on God”, “As many as I can support”, “I don’t know”.

**Table 3:** Association between female sterilization use and women’s socio-demographic characteristics

Characteristic	Frequency	Sterilized		***P***- value
		Not sterilized (%)	Sterilized (%)	
**Age**				
Younger than 30 years	11,000	100.0	0.0	< 0.001
30 years and older	7369	95.4	4.6	
**Marital status**				
Never in Union	4783	99.9	0.1	< 0.001
Married	11,000	97.3	2.7	
Formerly married	2500	98.8	1.2	
**Education level attained**				
No education	1781	96.8	3.2	< 0.001
Primary	11,000	97.9	2.1	
Secondary+	6095	99.1	1.0	
**Wealth quintile**				
Poor	6643	98.1	1.9	0.529
Middle	3460	98.0	2.0	
Rich	8403	98.3	1.7	
**Place of residence**				
Urban	4943	98.5	1.5	0.120
Rural	14,000	98.0	2.0	
**Employment status**				
Not employed	4986	99.1	0.9	< 0.001
Employed	14,000	97.8	2.2	
**Total number of children ever born**				
0–3 children	12,000	99.8	0.2	< 0.001
4+ children	6995	95.4	4.6	
**Ideal number of children**				
None	148	98.6	1.4	< 0.001
1–3 children	2937	99.5	0.5	
4+ children	15,000	98.0	2.0	
Non-numeric	437	95.6	4.4	
**Ideal number of boys**				
None	2278	98.8	1.2	< 0.001
1–3 boys	14,000	98.3	1.7	
4+ boys	1658	96.3	3.7	
Non-numeric	529	96.2	3.8	
**Ideal number of girls**				
None	2250	98.9	1.2	< 0.001
1–3 girls	14,000	98.4	1.7	
4+ girls	2012	96.6	3.4	
Non-numeric	529	96.2	3.8	
**Whether respondent earns more than husband/partner**				
Earn more than him	671	95.0	5.0	0.005
Earn less than him	5244	97.5	2.5	
Earn about the same	925	98.0	2.0	
Don’t know	256	97.0	3.0	
**Decision maker on respondent’s earnings**				
Respondent	3733	97.5	2.5	0.550
Husband and wife	2719	97.3	2.7	
Husband/Partner	644	96.6	3.4	
**Decision-maker on contraception**				
Respondent	1340	93.7	6.4	0.078
Husband/Partner	312	89.6	10.4	
Joint decision	2721	93.1	6.9	
**Knowledge of any family planning methods**				
No knowledge	187	100.0	0.0	0.140
Has knowledge	18,000	98.1	1.9	

### Predictors of female sterilization uptake

Table 4 presents predictors of female sterilization. Older women (> 30 years) are more likely to undergo sterilization compared to those less than 30 years. The odds of being sterilized were highest among women above 30 years (OR = 34.49, 95% CI = 13.33–99.88). On the other hand, women’s highest education level attained, place of residence, employment status, the ideal number of children and ideal number of sons preferred did not have a significant influence on female sterilization uptake. The findings also revealed that being from middle households reduced the odds of being sterilized as compared to being from the poor households. The odds of being sterilized of women from middle households were approximately 0.65 times compared to their counterparts. Furthermore, the results show that the odds of being sterilized for women who reported that the contraception decision-maker was the husband/partner were 2.42 times those of their counterparts who took independent decisions (OR = 2.42, 95% CI = 1.55–3.78). Women who took joint decisions with their husbands/partners had 1.38 odds higher of undergoing sterilization compared to those that took independent decisions (OR = 1.38, 95% CI = 1.02–1.86). Education level, place of residence, employment status, ideal number of children, and ideal number of boys were not significant.

**Table 4:** Complementary log-log model of predictors of female sterilization uptake

Characteristic	OR	***P***-value	95%CI
**Age**			
Below 30	1.00		
30+	36.49	***< 0.001***	13.33–99.88
**Education level**			
None	1.00		
Primary	0.91	0.554	0.66–1.25
Secondary+	0.69	0.102	0.44–1.08
**Wealth status**			
Poor	1.00		
Middle	0.65	***0.014***	0.47–0.92
Rich	0.74	0.073	0.53–1.03
**Place of residence**			
Urban	1.00		
Rural	0.83	0.329	0.57–1.20
**Employment status**			
Not employed	1.00		
Employed	0.98	0.914	0.66–1.45
**Total number of children ever born**			
0–3 children	1.00		
4+ children	3.19	***0.001***	1.63–6.22
**Contraception decision-maker**			
Respondent	1.00		
Husband/partner	2.42	***< 0.001***	1.55–3.78
Joint	1.38	***0.034***	1.02–1.86
**Ideal number of children**			
None	1.00		
1–3 children	0.29	0.151	0.06–1.56
4+ children	0.57	0.471	0.13–2.60
Non-numeric	0.35	0.420	0.03–4.54
**Ideal number of boys**			
None	1.00		
1–3 boys	1.14	0.578	0.72–1.79
4+ boys	1.34	0.266	0.80–2.26
Non-numeric	4.34	0.153	0.58–32.68

*CI* Confidence interval, *OR* Odds ratio

## Discussion

This study assessed factors influencing female sterilization uptake in Uganda. The study findings indicated a low uptake of female sterilization in Uganda. Female sterilization uptake was significantly associated with older age, middle wealth status, number of children ever born and joint decision making. The odds of taking up sterilization were highest among older women (> 30 years). This is not surprising as younger women may prefer reversible methods that are suitable for spacing over those that limit childbirths. Older women are more likely to be multiparous and prefer to use long acting and permanent methods as compared to younger women. The uptake of female sterilization may be affected by issues of informed choice and availability of quality services as was reported in a study conducted in India that women did not generally opt for sterilization in equal proportion due to very poor informed choice among women [21]. None the less, these findings are consistent with evidence from a similar study in Ethiopia [22] which revealed that older women (35–49 years) had three times higher odds of using sterilization contraception than their younger counterparts. The findings also partly agree with those of a study in Nigeria on female surgical sterilization which found that more than half of the women who opted for sterilization were aged between 35 to 39 years [23]. However, the study disagrees with findings of a study done in India where sterilization method uptake and acceptance was at a much younger age (mean age 28.9 years) among women [24].

The number of children ever born was associated with female sterilization uptake. This is probably because contraception choices are based on the already achieved fertility. Since sterilization is effective and irreversible, women who have not yet attained their desired fertility are less likely to use it. Women can confidently make contraceptive choices and implement decisions such as undergoing sterilization after achieving a certain number of children. This finding may also explain the high fertility preferences among women in Uganda. In addition, for some high parity women, these may be influenced by birth related experiences to stop childbearing and thus opt for sterilization. It is perhaps not very surprising since most users of female sterilization are those who do not wish to have any more children. Furthermore, women prefer many children as a source of workforce since Uganda is largely an agrarian economy with a poorly mechanized sector compared to other developed countries. This is in agreement with study findings from Uganda, rural Rakai which revealed that women with higher number of children had a significant desire to undergo sterilization [25] and partly agree with those from Brazil and India where women with three or more living children were more likely to undergo sterilization than their counterparts who had fewer children [26]. The finding is also consistent with a study done in Malawi [27] and another one in Nigeria where higher rates of sterilization uptake were found among women of parity four and above compared to those women with lower parities [28] but partly disagrees with findings in Nepal where intention to undergo sterilization was higher among women with a lower number of children [29].

The findings indicated that the contraceptive decision maker was significantly associated with the uptake of female sterilization. This is in agreement with previous studies done in other countries and contexts. For instance, in Zambia, joint contraceptive decision making between spouses was found to be a key determinant of uptake of injectable, long acting and permanent methods [30]. Similarly, in Ethiopia, women who discussed with their husbands about modern contraceptives in a bid to make contraceptive decisions were seven times more likely to use modern contraceptive methods than women who did not discuss at all [31] while in India, women who made joint decisions with their husbands were more likely to use sterilization compared to their counterparts who made independent decisions [32]. Eliason et al.(2014) asserted that women whose partners approved as decision makers of modern family planning were more likely to use modern contraceptives than their counterparts whose partners did not approve [33]. Likewise, in Bangladesh, there were higher odds of female sterilization acceptance among women whose partners approved compared to those whose partners did not approve [34]. Kabagenyi et al., (2014) found that the major barrier to use modern contraceptive methods among women in Uganda was partner disapproval [6].

This study also revealed that women who were from middle wealth households had reduced odds of being sterilized compared to their poor counterparts. Women from poor wealth class have higher birth rates and are more likely to undergo sterilization compared to women from middle wealth class. Also, women in middle wealth class enter marriages late and childbearing is relatively delayed compared to women from poor households who enter marriages early. This agrees with findings of a study conducted in India on dominance of sterilization and alternative choices of contraception which revealed that women from poor households relied on sterilization compared to their counterparts from rich households [32]. This study disagrees with findings of study done in Zambia where women from richer households had higher odds of using long acting and permanent methods compared to their counterparts from poorest households [30] and those on modern contraceptive use in Nigeria which reported that poorest women least used modern contraceptives compared to richest women [35].

Husband/partners’ decision on contraception was also associated with female sterilization uptake. Women who reported that the contraceptive decision maker was husband/partner had higher odds of utilizing female sterilization compared to those who took an independent decision. This can be attributed to the patriarchal nature of our society. This points to the significant influence of husbands/partners in determining women’s contraceptive choices. Women have limited decision-making power even on issues concerning their own health as a result of power dynamics from the male dominated societies. This is closely related to limited decision-making power among most women in many African societies. In addition, men have control over resources such as money and determine how they are used including facilitating a woman to undergo sterilization or not. Our findings are in agreement with the assertion that women whose partners are decision makers of modern family planning more likely to use modern contraceptives than their counterparts whose partners did not approve [6, 33, 34]. Similarly, study findings from Zambia [30], Ethiopia [31] and India [32] indicated that joint contraceptive decision making between spouses was a key determinant of uptake of family planning methods including female sterilization.

Although a woman’s highest level of education has been found to significantly affect her contraceptive choice and decision, our findings revealed that education attained did not have a significant association with the uptake of female sterilization. This is partly in disagreement with a study in Ethiopia which revealed that women with secondary level education had higher odds of using sterilization compared to those with no education [36]. The findings also partly contrast study findings in Nepal where it was revealed that an increase in a woman’s education reduced the probability that she will choose sterilization contraception [29]. The study also disagrees with study findings in India which revealed that women with secondary or higher education preferred temporary methods of contraception over sterilization [32] and in Ghana where women with no education were significantly less likely to use modern contraceptive including sterilization as compared to women with secondary or higher levels of education [33].

Our findings on place of residence partly disagree with other studies which indicated that place of residence significantly influenced contraceptive uptake including female sterilization. For instance, our study partly disagrees with findings from a comparative study between India and Brazil which indicated that female sterilization was generally higher among women in the rural areas compared to women in urban areas [26]. The results also disagree with a studies done in Uganda [37] and Nigeria [35] which reported that women in urban areas utilized modern contraceptives more than their counterparts in rural areas.

In this study, we found no evidence to suggest that women’s employment status associated with uptake of female sterilization. This is in contrary with the assertion that women’s employment status has a significant positive relationship with contraceptive use. Previous studies in India [32], Bangladesh [34, 38] asserted that women who are in paid employment were more likely to use sterilization than their counterparts who were unemployed because they are empowered to make independent reproductive health choices.

Our findings indicated that fertility preferences (ideal number of children and ideal number of boys) were not associated with the uptake of female sterilization by women of reproductive age. This finding partly disagrees with those of earlier studies such as in Uganda where contraceptive use among women is partly hindered by patriarchal family units that highly value children and encourage large family sizes [6], Ethiopia where the desire for no more children by women was significantly associated with women’s demand for long acting and permanent methods in [39], Zambia where women whose husbands desired more children were less likely to use modern contraceptive methods including female sterilization [30] and Pakistan where permanent method use among women was significantly associated with the number of sons they had ever born [40].

### Study limitations

Like other cross-sectional studies, the study could not determine a causal inference about female sterilization uptake. The study was also not able to clearly determine the causes of choice to undergo sterilization by individual women. In addition, as this study was based on secondary data, we were not able to investigate all factors associated with the uptake of female sterilization. In addition, this study is unable to explain the factors associated with family planning choices. A qualitative inquiry would provide a detailed understanding and explanation of the circumstances around uptake or no uptake of female sterilization as well as other family planning choices. Also, the other limitation of this study is that it did not include male sterilization (vasectomy) which is one of poorly utilized contraceptive method in Uganda. The study thus recommends that a similar study to investigate factors influencing the uptake of voluntary male sterilization be done.

## Conclusion and implications

The findings indicate that uptake of female sterilization in Uganda is generally low. The uptake was associated with being older, having ever given birth to at least four children and making independent contraceptive decisions. Therefore, there is need for male engagement by all stakeholders and implementing partners in matters related to women’s sexual and reproductive health, since it is evident that they play a key role in contraceptive decision making of service uptake.

The government of Uganda and its implementing partners of sexual and reproductive health programmes need to target higher parity (4+ children) and older (> 30 yrs) women through awareness creation campaigns and comprehensive counseling to promote female sterilization method acceptance. Also, promotion of permanent methods should target all sexually active young women to help them reach appropriate decisions on their contraceptive choices and limit their fertility if they desire to do so.

## Data Availability

The dataset used for this study are publicly available through the link https://dhsprogram.com/data/available-datasets.cfm.

## Abbreviations

UDHS: Uganda Demographic and Health Surveys
UBOS: Uganda Bureau of Statistics
IUD: Intra Uterine device
LAPM: Long acting and permanent methods
CI: Confidence Interval
OR: Odds Ratios
SSA: Sub Saharan Africa
